# 1,25-Dihydroxyvitamin D_3_ Restrains CD4^+^ T Cell Priming Ability of CD11c^+^ Dendritic Cells by Upregulating Expression of CD31

**DOI:** 10.3389/fimmu.2019.00600

**Published:** 2019-03-28

**Authors:** Louise Saul, Iris Mair, Alasdair Ivens, Pamela Brown, Kay Samuel, John D. M. Campbell, Daniel Y. Soong, Nadine Kamenjarin, Richard J. Mellanby

**Affiliations:** ^1^MRC Centre for Inflammation Research, Queen's Medical Research Institute, University of Edinburgh, Edinburgh, United Kingdom; ^2^Ashworth Laboratories, Centre for Immunity, Infection and Evolution, University of Edinburgh, Edinburgh, United Kingdom; ^3^Biomolecular Core, MRC Centre for Reproductive Health, Queen's Medical Research Institute, Shared University Research Facility, University of Edinburgh, Edinburgh, United Kingdom; ^4^Scottish National Blood Transfusion Service, Edinburgh, United Kingdom; ^5^MRC Centre for Reproductive Health, Queen's Medical Research Institute, Edinburgh, United Kingdom; ^6^Easter Bush Veterinary Centre, Hospital for Small Animals, The Roslin Institute, The Royal (Dick) School of Veterinary Studies, The University of Edinburgh, Roslin, United Kingdom

**Keywords:** dendritic cells, vitamin D, 1, 25(OH)_2_D_3_, autoimmune disease, CD31, T cell priming

## Abstract

Dendritic cells (DC) are specialized sentinel cells that bridge the innate and adaptive immune response and play a crucial role in shaping the adaptive immune response. Vitamin D, a known epidemiological risk factor for the development of several autoimmune diseases, influences the development of dendritic cells. Consequently, vitamin D metabolites are frequently used in protocols to develop therapeutic dendritic cell therapies for autoimmune diseases. However, the mechanisms by which vitamin D modulates DC function remain poorly understood. We investigated the effects of vitamin D on murine CD11c^+^ bone marrow derived DC (BMDC) function by analyzing global gene expression in CD11c^+^ BMDC generated in the presence (VitD-CD11c^+^BMDC) or absence (Veh-CD11c^+^BMDC) of the active vitamin D metabolite, 1,25-dihydroxyvitamin D_3_ (1,25(OH)_2_D_3_). Seven genes were significantly increased in expression in both immature and LPS-matured VitD-CD11c^+^BMDC, one of which was CD31, a member of the immunoglobulin superfamily. Gene knockdown of CD31 enhanced the ability of VitD-CD11c^+^BMDC to prime naïve CD4^+^ T cells *in vitro*; conversely, increased expression of CD31 on vehicle treated CD11c^+^BMDC restrained their T cell priming abilities. Time-lapse imaging of BMDC and CD4^+^ T cells during *in vitro* priming revealed that CD31 reduced the BMDC–T cell interaction time. Finally, we confirmed a similar effect of 1,25(OH)_2_D_3_ on human CD34^+^ cell-derived CD11c^+^DC, whereby DC generated in the presence of 1,25(OH)_2_D_3_ had increased CD31 expression. In summary, we show that both mouse and human DC generated in the presence of 1,25(OH)_2_D_3_ upregulate CD31 expression, resulting in a reduced ability to prime CD4^+^ T cells by impairing a stable cell-cell contact.

## Introduction

Dendritic cells (DC) are professional antigen presenting cells which play a crucial role in shaping the adaptive immune response (1). They have the ability to either tolerize or activate T cells depending on their activation status and concomitant expression of co-stimulatory or inhibitory molecules. In the absence of activation, antigen presentation by steady-state DC can lead to T cell unresponsiveness and tolerance (1). In the presence of co-stimulatory molecules, antigen presentation by DC results in T cell activation.

A wide range of factors can influence the phenotype and function of DC. One of the most widely studied molecules known to alter the development of DC is the active vitamin D metabolite, 1,25-dihydroxyvitamin D_3_ (1,25(OH)_2_D_3_) (2– 5). The interest in probing the effects of vitamin D on DC function has, in part, been due to the wealth of data which now links suboptimal vitamin D status to the development and progression of many autoimmune diseases (6–8). Several groups have demonstrated that DC generated in the presence of 1,25(OH)_2_D_3_ from either bone marrow cells or monocytes have a reduced ability to prime T cells *in vitro* and in many experimental systems can tolerize T cells (9–12). These findings have led to the development of clinical trials of tolerogenic 1,25(OH)_2_D_3_ conditioned DC in human patients with autoimmune conditions such as rheumatoid arthritis and multiple sclerosis (5, 13–15).

However, the mechanisms by which 1,25(OH)_2_D_3_ manipulates the phenotype of DC remain incompletely understood. We, and others, have shown that the addition of 1,25(OH)_2_D_3_ to bone marrow cell cultures leads to the generation of BMDC which have lower MHC class II expression alongside reduced expression of co-stimulatory molecules such as CD80 and CD86 (16, 17). Given the widespread impact that 1,25(OH)_2_D_3_ can have on immune cells, it would appear likely that additional co-stimulatory or inhibitory pathways may be influenced by exposure to 1,25(OH)_2_D_3_. To explore this further we performed a global gene expression analysis on CD11c^+^BMDC generated in the absence (Veh-CD11c^+^BMDC) or presence of 1,25(OH)_2_D_3_ (VitD-CD11c^+^BMDC). We focused our attention on CD11c^+^ cells for two key reasons; firstly, CD11c^+^ cells are known to have potent antigen presenting capacity and secondly, the addition of 1,25(OH)_2_D_3_ is known to lower the proportion of CD11c^+^ in murine BMDC cultures (16, 17). Consequently, we wanted to evaluate gene expression in cells which have the capacity to prime antigens and did not want to confound our data by including cells which were CD11c^−^ and did not express MHC class II molecules.

Here, we present microarray results on this defined population which demonstrate that the addition of 1,25(OH)_2_D_3_ resulted in a large number of differentially expressed genes. Specifically, we discovered that CD31 was one of only seven genes whose expression was upregulated in both immature and LPS-matured VitD-CD11c^+^BMDC. CD31 is a 130-kDa member of the immunoglobulin superfamily, a single-chain transmembrane glycoprotein with six C2-type Ig-like extracellular domains, and a cytoplasmic tail containing two immunoreceptor tyrosine-based inhibitory motifs (ITIMs) (18, 19). CD31 is concentrated at endothelial tight junctions where it supports endothelial cell layer integrity (20), and is also expressed at lower levels on platelets and most leukocytes (21). CD31 mostly facilitates cell-cell adhesion via trans-homophilic interactions (22, 23), but has also been reported to interact in a heterophilic manner via CD177 (24), αvβ3 (25), glycosaminoglycans (26), and CD38 (27). Not surprisingly, CD31 has been implicated in mediating leukocyte migration across the endothelial cell layer (28), but has also drawn attention as a potential immunomodulatory molecule important for communication between immune cells, e.g., as a detachment signal between live neutrophils and macrophages (29), and as a co-inhibitory molecule on T cells (21) and DC (30). Very little is known about the factors which regulate CD31 expression in immune cells.

Here, we present data revealing 1,25(OH)_2_D_3_ as a potent inducer of CD31 expression on BMDC, and identify increased CD31 levels on BMDC as a novel mechanism by which 1,25(OH)_2_D_3_ restrains the ability of BMDC to prime naïve CD4^+^ T cells.

## Materials and Methods

### Mice, Antigens, and Tissue Culture Medium

B10.PLxC56BL/6 (CD45.2) and Tg4 (CD45.1) mice were bred under specific pathogen-free conditions at the University of Edinburgh. All experiments had local ethical approval from the University of Edinburgh's Animal Welfare and Ethical Review Body and were performed in accordance with UK legislation. All mice used in the experiments reported were female as this allowed for standardization of experiment groups and permitted the housing of mice from different litters in the same experimental cage. The mice were maintained in individually ventilated cages, and were between 8 and 12 weeks old when used for experiments. The housing facility was compliant with Federation of European Laboratory Animal Science Associations guidelines on screening mice for infectious diseases. Tg4 mice express a transgenic T cell receptor (TCR) recognizing the Ac1-9 peptide of myelin basic protein (MBP) in association with I-A^u^ (31). The MBP Ac1-9 (4Tyr) analog peptide was obtained from Cambridge Research Biochemicals (Teesside, UK). To obtain cell culture medium, RPMI 1640 medium was supplemented with 2 mM L-glutamine, 100 U/ml penicillin, 100 μg/ml streptomycin, and 5 × 10^−5^ M 2-ME (all from Gibco, Paisley, UK) and 10% heat-inactivated FCS (Labtech, East Sussex, UK).

### Generation and Stimulation of Murine BMDC

BMDC were generated in the presence of recombinant GM-CSF (Peprotech, London, UK) for 9 days as previously described (32). Briefly, bone marrow was collected from femurs and tibias of B10.PLxC57BL/6 mice, and clusters within the bone marrow suspension were dispersed by vigorous pipetting. Red blood cells were lysed with RBC lysis buffer (Sigma-Aldrich, Dorset, UK) for 1 min and cells subsequently washed once in cell culture medium. Cells were seeded into the center of 6 well plates at 2 × 10^5^/ml in 2 ml of cell culture medium with the addition of 20 ng/ml GM-CSF. At day 3, a further 2 ml of cell culture medium containing 20 ng/ml GM-CSF was added to each well. On day 6, 2 ml of culture supernatant was removed and replaced with 2 ml fresh culture medium containing 20 ng/ml GM-CSF. Vehicle (100% ethanol) or 1,25(OH)_2_D_3_ was added to the BMDC culture media at the concentration indicated in the text initially and at all subsequent media changes. At day 9, the BMDC cells were harvested and CD11c^+^ cells were isolated by FACS (Supplementary Figure 1). The CD11c^+^ BMDC were re-plated at 2 × 10^6^ BMDC/ml in cell culture medium, 5 ng/ml of GM-CSF with 0.1 μg/ml Ac1-9(4Tyr) MBP for an additional 18 h. 0.1 μg/ml lipopolysaccharide (LPS) was added to some overnight CD11c^+^ BMDC cultures (Sigma-Aldrich, Dorset UK). No 1,25(OH)_2_D_3_ was added to the culture media during the overnight stimulation with LPS and MBP. Cytokines were measured in BMDC supernatants by Ready-SET-Go ELISA as per manufacturer's instructions (eBioscience, San Diego, USA).

### Generation of Human DC From Mobilized Peripheral Blood CD34^+^ Progenitor Cells

CD34^+^ cells were obtained from a commercial source (AllCells, LLC., Alameda, CA, USA). The CD34^+^ cells had been isolated by positive selection with an immunomagnetic bead system, from volunteer mobilized peripheral blood CD34^+^ stem/progenitor cell donations following informed consent. CD34^+^ progenitors were cultured in Iscove Basal Medium (Biochrom) with BIT 9500 Serum Substitute (Stemcell Technologies), 100 ng/ml SCF (Pharmacy), 100 ng/ml FLT3L (Peprotech), and 50 ng/ml TPO (Peprotech). Medium was replenished every 4 days by addition to cultures and splitting into fresh flasks to maintain optimum culture conditions.

After 3 weeks, expanded cells were differentiated to DC by culture in TexMACS medium (Miltenyi Biotech) supplemented with 50 ng/ml GM-CSF (Peprotech) and 15 ng/ml IL-4 (Peprotech), in the presence of 20 nM 1,25(OH)_2_D_3_ or vehicle (100% ethanol). Seven days later, cells were analyzed by flow cytometry.

### RNA Extraction and Microarray Analysis of BMDC

RNA was extracted from CD11c^+^BMDC using commercially available kits (RNeasy Mini Kit). RNA integrity number (RIN >8) was assessed using the 2100 Bioanalyser and RNA 6000 Pico kit (Agilent, CA, USA). Sense strand cDNA preparation from RNA samples was generated and labeled using Ambion WT expression kit (Life Technologies, Paisley, UK) and Affymetrix Gene Chip-WT terminal labeling kit according to manufacturer's instructions. Samples were hybridized to Affymetrix Mouse Gene 2.1 ST 16-Array Plate (Affymetrix, CA, USA). Raw data was normalized using robust multichip average method and principal component analysis created using Genomics suite (Partek Incorporated, MO, USA).

### Primary Tg4 CD4^+^ T Cell Activation Assays

To study the primary activation of Tg4 CD4^+^ T cells, varying numbers (as stated) of BMDC (CD45.2) were cultured with 2 × 10^4^ Tg4 CD4^+^ T cells (CD45.1) per well in a round bottomed, 96 well plate. The CD4^+^ T cells were purified using microbeads as per manufacturer's instructions (Miltenyi Biotech, Surrey, UK). Tg4 CD4^+^ T cell production of cytokines (IL-2, TNF-α, GM-CSF, and IFN-γ) was assessed in culture supernatants by Ready-SET-Go ELISA (eBioscience, San Diego, USA). IL-2 was measured in supernatants after 48 h of culture and IFN-γ, TNF-α, and GM-CSF were measured after 72 h of culture.

### Lentiviral Transduction of BMDC for Modulation of CD31 Expression

To investigate the effects of CD31 expression on the ability of CD11c^+^BMDC to prime CD4^+^ T cells, lentiviral constructs expressing GFP and murine CD31 or murine CD31 siRNA were developed. For the CD31 overexpression construct (pLenti6-cppt-CMV-mCD31-IRES-emGFP-opre), murine platelet/endothelial cell adhesion molecule-1 (Pecam-1; mCD31) isoform 3 (NM_001305157.1) was synthesized by Integrated DNA Technologies (IDT, Leuven, Belgium). The synthetic gene was flanked by *att*B1 and *att*B5r GATEWAY cloning sites (Thermo Fisher Scientific: Invitrogen UK) and a Kozak consensus overlapped the start codon. Using GATEWAY technology this was used to create the lentiviral shuttle vector pLenti6-cppt-CMV-mCD31-IRES-emGFP-opre (33). Negative control lacked mCD31. By partially concentrating lentivirus in Optimem (Invitrogen, UK) titers of 10^7^ transduction units/ml (TU/ml) were obtained.

For siRNA-expressing vectors, knock down target sequences within mCD31 were identified by a combination of literature searches and the output of the Invitrogen Block-iT RNAi designer web program. A scrambled sequence was used as a negative control. The synthesized oligonucleotides were annealed, then inserted into pcDNA6.2-GW_emGFP-miR 285, 816, and 1827, with numbers identifying the target distance from the start codon of mCD31 (Table 1). These were shuttled into pLenti6.2-cppt-CMV-DEST-opre using GATEWAY (Invitrogen, UK), and packaged. Titers of 10^8^ TU/ml were routinely obtained.

**Table 1:** Synthetic oligonucleotides used for miR.

**miR RNAi**	**Name**	**DNA Sequence (5^**′**^-3^**′**^)**
Neg	Scr top	TGCTGAAATGTACTGCGCGTGGAGACGTTTTGGCCACTGACTGACGTCTCCACGCAGTACATTT
	Scr bottom	AAATGTACTGCGTGGAGACGTCAGTCAGTGGCCAAAACGTCTCCACGCGCAGTACATTTCCAGCA
CD31	285 top	TGCTGATCACTGTGCATTTGTACTTCGTTTTGGCCACTGACTGACGAAGTACATGCACAGTGAT
	285 bottom	CCTGATCACTGTGCATGTACTTCGTCAGTCAGTGGCCAAAACGAAGTACAAATGCACAGTGATC
	816 top	TGCTGTAGACAGCTTCACTGCTTTGCGTTTTGGCCACTGACTGACGCAAAGCAGAAGCTGTCTA
	816 bottom	CCTGTAGACAGCTTCTGCTTTGCGTCAGTCAGTGGCCAAAACGCAAAGCAGTGAAGCTGTCTAC
	1827 top	TGCTGTTCCTCAGGAAGTAGCATTTGGTTTTGGCCACTGACTGACCAAATGCTTTCCTGAGGAA
	1827 bottom	CCTGTTCCTCAGGAAAGCATTTGGTCAGTCAGTGGCCAAAACCAAATGCTACTTCCTGAGGAAC

Six days following BMDC induction, cells were seeded at 5 × 10^5^ cells/well in a 24 well cell culture dish in 1 ml cell culture medium and lentiviral constructs added at an MOI of 20. For CD31 overexpression lentiviral constructs, 6 μg/ml DEAE were added to enhance transduction efficiency. Two days later, BMDC were stimulated for 18 h as described above and CD11c^+^GFP^+^ BMDC isolated by FACS prior to further experimentation.

### Preparation of Spleen Mononuclear Cells and FACS Analysis

Single cell suspensions were made from the spleen and draining lymph nodes, red blood cells were lysed using an ammonium chloride buffer (Sigma Aldrich, Dorset, UK), and cells were then re-suspended in FACS buffer (PBS, 2% fetal calf serum, 0.01% sodium azide (Sigma Aldrich, Dorset, UK). Fc receptors were blocked with supernatant from the hybridoma 2.4G2. All antibodies were from eBioscience, Hatfield, UK, except where stated; live/dead fixable cell stain conjugated to ef455 (Life Technologies), anti-CD4-APC, anti-CD4-AF700 (BD Pharmingen, Oxford, UK), anti-CD11c-PE-Cy7, anti-CD11c-ef450, anti-Ki67-PE-Cy7, anti-CD11b-Af700, anti-CD45.1-FITC, anti-CD44-APC-Cy7, anti-CD80-PE, anti-CD86-APC, anti-CD62L-PerCP-Cy5.5, and anti-Foxp3-ef450. FACS data were collected using a 6 laser LSR Fortessa (BD Biosciences, New Jersey, USA) and analyzed using FlowJo software (Tree Star, Olten, Switzerland).

### PrimeFlow RNA Assay

Murine BMDC were stained with fixable viability dye for 30 min at 4°C, washed once, and stained for surface markers of interest (anti-CD11c-PE-Cy7, anti-CD31-PE, anti-CD86-bv785, and anti-MHC-II-PE-ef610) for 30 min at 4°C, followed by another washing step. PrimeFlow RNA assay was performed for CD31 mRNA (VB4-10932) as per manufacturer's instructions (Affymetrix). Briefly, cells were fixed in PrimeFlow RNA Fixation Buffer 1 for 30 min at 4°C, then washed twice in PrimeFlow RNA Permeabilization Buffer supplemented with RNAse Inhibitors, and fixed in PrimeFlow RNA Fixation Buffer 2 for 60 min at room temperature. Following two washing steps with PrimeFlow Wash Buffer, target probe hybridization was performed for 2 h at 40°C. Samples were washed twice again in PrimeFlow RNA Wash Buffer and stored overnight in PrimeFlow RNA Wash Buffer supplemented with RNAse Inhibitor 1. The following day, consecutive signal amplification steps were performed according to manufacturer's instructions at 40°C, prior to sample acquisition on a BD LSRFortessa cell analyzer (BD biosciences).

### Time-Lapse Imaging

BMDC transduced with control or CD31 overexpressing lentiviral vector were stimulated for 4 h with 0.1 μg/ml LPS and 0.1 μg/ml 4Y peptide. CD11b^+^CD11c^+^GFP^+^ cells were FACS sorted and re-plated onto poly-L-lysine coated glass-bottom plates at a density of 3 × 10^4^ cells/ml in cell culture medium. After overnight adhesion, BMDC were carefully washed twice in PBS before naïve CD4^+^ T cells from the spleen of a Tg4 (CD45.1) mouse were slowly added at a 4:1 ratio in phenol red-free HBSS. Brightfield images (5 randomly selected fields in each well) were acquired every 30 s using an Axiovert 200 microscope in a CO_2_-supplemented chamber at 37°C. Movies of cells were tracked in Fiji ImageJ to determine the duration of interaction between BMDC and CD4^+^ T cells.

### Statistical Analysis

For the bioinformatic analyses, a total of 20 arrays (*n* = 3 Veh-CD11c^+^BMDC no LPS, *n* = 3 VitD-CD11c^+^BMDC no LPS, *n* = 6 Veh-CD11c^+^BMDC LPS, *n* = 8 VitD-CD11c^+^BMDC LPS) were assessed for quality control using the arrayQualityMetrics package in Bioconductor (www.bioconductor.org). log_2_ expression values for the high quality arrays were quantile normalized. Pairwise group comparisons were undertaken using linear modeling with the limma package in Bioconductor. Subsequently, empirical Bayesian analysis was applied, including vertical (within a given comparison) *p*-value adjustment for multiple testing, which controls for false-discovery rate. An adjusted *p*-value of < 0.05 was applied as the significance threshold. Functional enrichment analyses of KEGG pathways associated with the significant loci were performed using hypergeometric tests (34). Focused genes of interest lists were assembled from the literature and other publicly available resources. Statistical analysis of cytokine results was performed using a two tailed Student's *t*-test. Cytokine concentrations are presented as mean concentration ±SEM. The proportion of T cell and BMDC interactions which were < 5 min was compared between Veh-CD11c^+^BMDC and VitD-CD11c^+^BMDC by a Fisher's exact test. Significance was set at *p* < 0.05.

## Results

### Addition of 1,25(OH)_2_D_3_ to BMDC Cultures Leads to Altered Gene Expression Profiles in CD11c^+^BMDC

**Figure 1:**
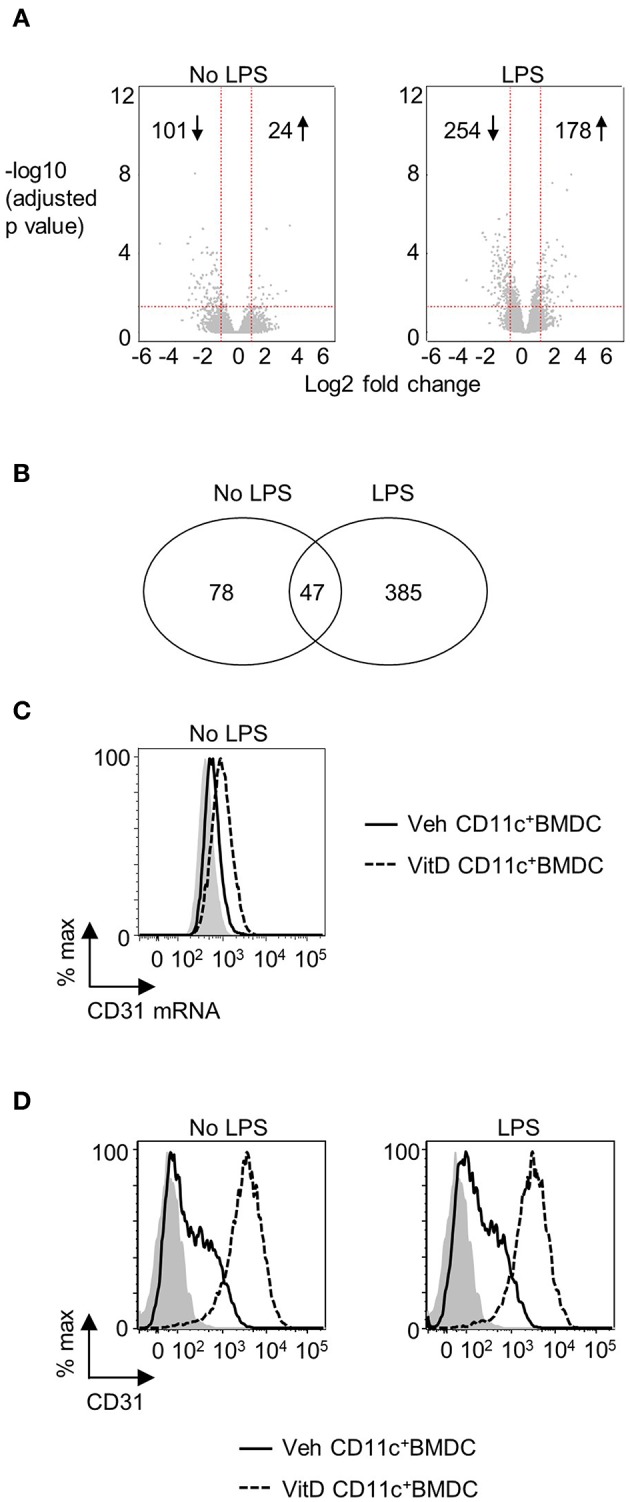
CD31 is upregulated by 1,25(OH)_2_D_3_ in BMDC. Bone marrow derived dendritic cells (BMDC) were generated over 9 days in the presence of 1,25(OH)_2_D_3_ (VitD) or absence (Veh) of 20 mM 1,25(OH)_2_D_3_. **(A)** Veh and VitD BMDC were matured for 18 h in the presence or absence of 0.1 μg/ml LPS. mRNA was extracted and microarray performed. Differential gene expression caused by 1,25(OH)_2_D_3_ in the absence (left) or presence of LPS stimulation (right) is depicted as volcano plots. **(B)** Number of genes differentially regulated by 1,25(OH)_2_D_3_ in CD11c^+^BMDC in the presence or absence of LPS stimulation. **(C)** Representative flow plot of CD31 mRNA levels in Veh (continuous line) and VitD (dashed line) CD11c^+^BMDC as assessed by PrimeFlow. Fluorescence minus one is indicated by gray shaded area. *n* = 3. **(D)** Representative flow plots of CD31 protein expression in Veh (continuous line) and VitD (dashed line) CD11c^+^BMDC in the absence (left panel) or presence (right panel) of LPS stimulation as assessed by flow cytometry. Isotype control is indicated by gray shaded area.

1,25(OH)_2_D_3_ and vehicle conditioned BMDC were generated as described in materials and methods. After 9 days of culture, CD11c^+^ cells were isolated by FACS and cultured overnight in 1,25(OH)_2_D_3_ free media in the presence or absence of LPS. Global gene expression analysis revealed that 101 genes were downregulated and 24 were upregulated in non-LPS stimulated VitD-CD11c^+^BMDC compared to Veh-CD11c^+^BMDC (Figure 1A). The number of genes differentially expressed in VitD-CD11c^+^BMDC increased following LPS stimulation, with 254 genes downregulated and 178 upregulated (Figure 1A). The genes with the greatest upregulation and downregulation in VitD-CD11c^+^BMDC compared to Veh-CD11c^+^BMDC are shown in Tables 2–5. The gene expression datasets for this study can be found in the Gene Expression Omnibus, accession number GSE114768.

**Table 2:** List of top 10 genes upregulated in VitD-CD11c^+^BMDC compared to Veh-CD11c^+^BMDC without LPS stimulation.

**Gene symbol**	**Description**	**EntrezID**	**Log FC**	**Adjusted *p*-value**
*Epb41l3*	Erythrocyte protein band 4.1-like 3	13823	3.5	3.80E-06
*Srgap3*	SLIT-ROBO Rho GTPase activating protein 3	259302	2	5.40E-06
*Adgre1*	EGF-like module containing, mucin-like, hormone receptor-like sequence 1	13733	1.5	0.0013
*Mmp8*	Matrix metallopeptidase 8	17394	2.3	0.0041
*Serpinb2*	Serine (or cysteine) peptidase inhibitor, clade B, member 2	18788	3.2	0.0081
*Stfa3*	Stefin A3	20863	2.7	0.011
*Tanc2*	Tetratricopeptide repeat, ankyrin repeat and coiled-coil containing 2	77097	1.8	0.012
*Pecam1*	CD31 antigen	18613	1.3	0.017
*S100a9*	S100 calcium binding protein A9 (calgranulin B)	20202	2	0.019
*Syne1*	Spectrin repeat containing, nuclear envelope 1	64009	2.4	0.019

**Table 3:** List of top 10 genes downregulated in VitD-CD11c^+^BMDC compared to Veh-CD11c^+^BMDC without LPS stimulation.

**Gene symbol**	**Description**	**EntrezID**	**Log FC**	**Adjusted p value**
*Ccl5*	Chemokine (C-C motif) ligand 5	20304	−3	1.40E-05
*Fabp4*	Fatty acid binding protein 4, adipocyte	11770	−5.1	3.10E-05
*Ifi27l2a*	Interferon, alpha-inducible protein 27 like 2A	76933	−1.3	9.00E-05
*Gbp5*	Guanylate binding protein 5	229898	−1.5	9.00E-05
*Gbp2*	Guanylate binding protein 2	14469	−1.6	9.00E-05
*Ace*	Angiotensin I converting enzyme (peptidyl-dipeptidase A) 1	11421	−2.3	9.00E-05
*Lilra5*	Leukocyte immunoglobulin-like receptor, subfamily A (with TM domain), member 5	232801	−1.7	0.00011
*Adam23*	A disintegrin and metallopeptidase domain 23	23792	−2.8	0.00021
*Lgals3bp*	Lectin, galactoside-binding, soluble, 3 binding protein	19039	−1.4	0.00039
*Slc35g1*	Solute carrier family 35, member G1	240660	−1.4	0.00059

**Table 4:** List of top 20 genes upregulated in VitD-CD11c^+^BMDC compared to Veh-CD11c^+^BMDC with LPS stimulation.

**Gene symbol**	**Description**	**EntrezID**	**Log FC**	**Adjusted *p*-value**
*Epb41l3*	Erythrocyte protein band 4.1-like3	13823	3	9.20E-09
*Srgap3*	SLIT-ROBO Rho GTPase activating protein 3	259302	1.8	2.50E-08
*Lifr*	Leukemia inhibitory factor receptor	16880	2.7	5.70E-08
*Angptl2*	Angiopoietin-like 2	26360	2.1	0.00002
*Adgre1*	EGF-like module containing, mucin-like, hormone receptor-like sequence 1	13733	1.4	0.000028
*Mmp8*	Matrix metallopeptidase 8	17394	2.4	0.000043
*Gstp1*	Glutathione S-transferase, pi 1	14870	1.5	0.000046
*Acpp*	Acid phosphatase, prostate	56318	1.3	0.000066
*Fam110c*	Family with sequence similarity 110, member C	104943	1.3	0.00012
*Ctla2b*	Cytotoxic T lymphocyte-associated protein 2 beta	13025	2.4	0.00012
*Nppc*	Natriuretic peptide type C	18159	2.6	0.00014
*Ppfibp2*	PTPRF interacting protein, binding protein 2 (liprin beta 2)	19024	1.2	0.00017
*Gja1*	Gap junction protein, alpha 1	14609	1.9	0.0002
*Ripk3*	Receptor-interacting serine-threonine kinase 3	56532	1	0.00022
*Hgf*	Hepatocyte growth factor	15234	2	0.00023
*B3glct*	Beta-3-glucosyltransferase	381694	1.3	0.00028
*Nucb2*	Nucleobindin 2	53322	0.8	0.0003
*Dapk2*	Death-associated protein kinase 2	13143	1.7	0.00042
*Mmp13*	Matrix metallopeptidase 13	17386	1.3	0.00079
*Il13*	Interleukin 13	16163	2.9	0.0011

**Table 5:** List of top 20 genes downregulated in VitD-CD11c^+^BMDC compared to Veh-CD11c^+^BMDC with LPS stimulation.

**Gene symbol**	**Description**	**EntrezID**	**Log FC**	**Adjusted *p*-value**
*Fyn*	Fyn proto-oncogene	14360	−1.8	1.70E-06
*Gpcpd1*	Glycerophosphocholine phosphodiesterase GDE1 homolog (*S. cerevisiae*)	74182	−1.1	5.90E-06
*Cnn3*	Calponin 3, acidic	71994	−2.8	8.80E-06
*Tmem176a*	Transmembrane protein 176A	66058	−2.0	1.00E-05
*Sesn3*	Sestrin 3	75747	−1.4	1.40E-05
*Mall*	Mal, T cell differentiation protein-like	228576	−2.7	1.40E-05
*Clu*	Clusterin	12759	−2.6	1.90E-05
*Hmgn3*	High mobility group nucleosomal binding domain 3	94353	−1.9	2.20E-05
*Net1*	Neuroepithelial cell transforming gene 1	56349	−1.8	4.50E-05
*Stk39*	Serine/threonine kinase 39	53416	−2.1	4.60E-05
*Bhlhe41*	Basic helix-loop-helix family, member e41	79362	−1.2	4.60E-05
*Fstl1*	Follistatin-like 1	14314	−1.4	6.70E-05
*Slc39a2*	Solute carrier family 39 (zinc transporter), member 2	214922	−1.9	7.00E-05
*Arap2*	ArfGAP with RhoGAP domain, ankyrin repeat, and PH domain 2	212285	−2.2	8.40E-05
*Serpinb9*	Serine (or cysteine) peptidase inhibitor, clade B, member 9	20723	−1.4	0.00012
*Plxnc1*	Plexin C1	54712	−1.8	0.00012
*Pvrl2*	Poliovirus receptor-related 2	19294	−1.1	0.00015
*Cd86*	CD86 antigen	12524	−1.1	0.00016
*Htra4*	HtrA serine peptidase 4	330723	−1.2	0.00016
*Dennd2a*	DENN/MADD domain containing 2A	209773	−1.6	0.00017

### CD31 Expression Is Increased on VitD-CD11c^+^BMDC

There were 47 genes which were differential expressed in VitD-CD11c^+^BMDC (in both immature and LPS-matured cells), of which 7 were upregulated and 40 were downregulated (Figure 1B). The 7 upregulated genes were *Pecam1, Cd300ld, Adgre1, Epb41l3, Srgap3, Klk1b1*, and *Mmp8*. The increased expression of CD31 in VitD-CD11c^+^BMDC was striking given recent studies which have indicated that CD31 plays an important role in regulating a wide range of leukocytes (30, 35). The increased mRNA expression of CD31 in non-LPS stimulated VitD-CD11c^+^BMDC was confirmed by flow cytometric evaluation of CD31 mRNA (Figure 1C). Protein expression of CD31 on VitD-CD11c^+^BMDC was subsequently examined by flow cytometry which demonstrated an increase in CD31 expression on BMDC which had been conditioned with 20 nM 1,25(OH)_2_D_3_ (Figure 1D). This increase was dependent on the dose of 1,25(OH)_2_D_3_, with sequential increases in CD31 expression occurring in CD11c^+^BMDC which had been generated in the presence of 5, 10, and 15 nM of 1,25(OH)_2_D_3_ (Supplementary Figure 2A). The increase in expression of CD31 on VitD-CD11c^+^BMDC was an early event with high levels of CD31 detectable on VitD-CD11c^+^BMDC as early as day 3 of the BMDC cultures (Supplementary Figure 2B). When 1,25(OH)_2_D_3_ was added to the BMDC culture on day 3 or day 6, rather than on day 0, CD31 levels were still upregulated on day 9, although at lower levels compared to BMDC which were cultured in the presence of 1,25(OH)_2_D_3_ since the beginning of the culture (Supplementary Figure 2C). However, when 1,25(OH)_2_D_3_ was added to or removed from Veh-CD11c^+^BMDC or VitD-CD11c^+^BMDC, respectively, during overnight stimulation with LPS, no change in CD31 levels was observed (Supplementary Figure 2D), indicating that CD31 levels are determined during the generation, not the maturation, of BMDC.

### CD31 Expression on VitD-CD11c^+^BMDC Restrains Their Ability to Prime CD4^+^ T Cells

**Figure 2:**
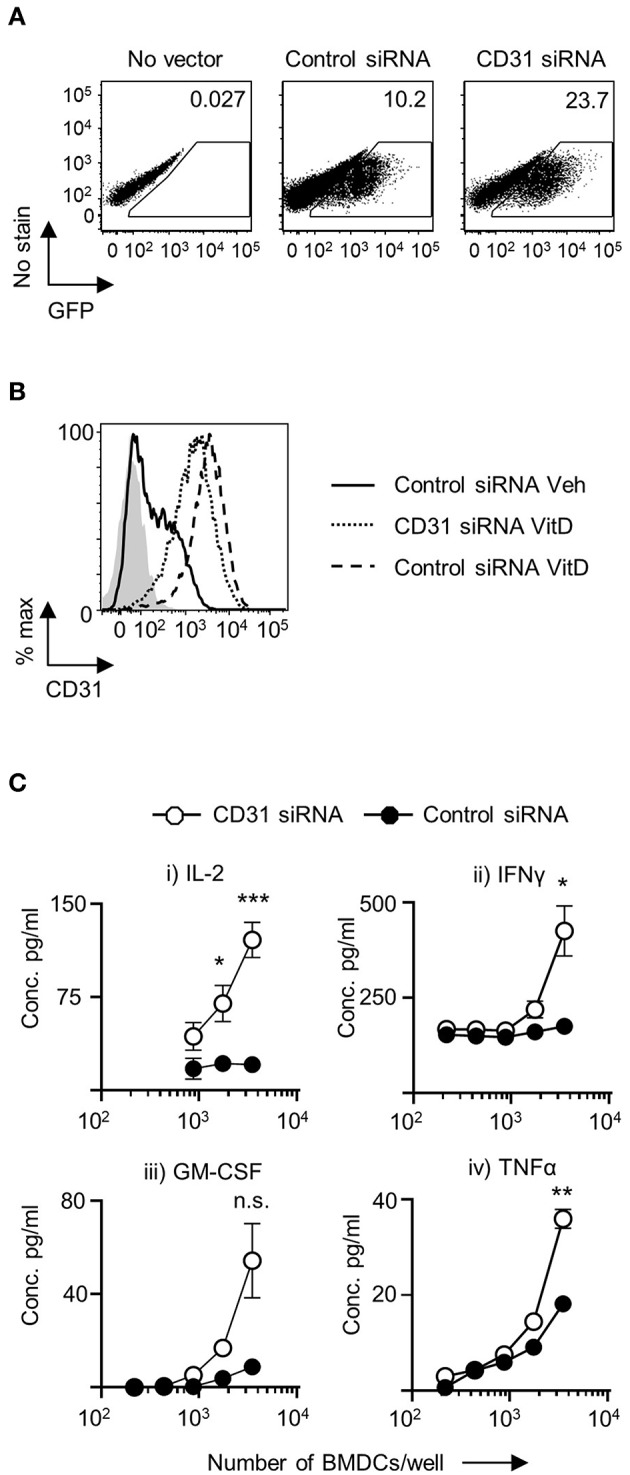
Downregulation of CD31 in VitD BMDC results in enhanced *in vitro* priming of CD4^+^ T cells. Bone marrow derived dendritic cells (BMDC) were generated over 9 days with 20 ng/ml GM-CSF in the presence (VitD) or absence (Veh) of 20 nM 1,25(OH)_2_D_3_. On day 6, BMDC were transduced with indicated lentiviral constructs, all expressing GFP. **(A)** Representative flow plots of transduction efficiency as determined by GFP expression, utilized for flow cytometric sorting of transduced cells for downstream analysis. **(B)** Representative flow plot of CD31 protein expression in VitD CD11c^+^BMDC transduced with a lentiviral vector containing CD31 siRNA (dotted line) compared to VitD and Veh CD11c^+^BMDC transduced with a lentiviral vector containing control siRNA (dashed line and continuous line, respectively). Isotype control is indicated by gray shaded area. **(C)** VitD CD11c^+^BMDC transduced with control siRNA (black) or CD31 siRNA expressing (white) lentiviral vector were matured in the presence of 0.1 μg/ml LPS for 4 h and pulsed with 0.1 μg/ml 4Y peptide, and were subsequently co-cultured at indicated cell numbers with 2 × 10^4^ Tg4 CD4^+^ T cells for 48 h (IL-2) or 72 h (all other cytokines). Cytokine release was assessed by ELISA. *n* = 3, representative of 2 experiments (^*^p < 0.05, ^**^p < 0.01, and ^***^p < 0.001).

CD31 is expressed on both naïve and activated CD4^+^ T cells (Supplementary Figure 3). Consequently, it would be challenging to dissect the role of CD31 expression in the ability of BMDC to prime CD4^+^ T cells *in vitro* by adding a blocking CD31 antibody to the BMDC-T cell co-cultures, as this would also affect CD31 availability on T cells. To precisely explore the role of CD31 expression in CD11c^+^BMDC function, we used lentiviral constructs to either upregulate or downregulate CD31 expression. Initially, we produced three siRNA lentivirus constructs. Both control vectors and siRNA constructs were effective in transducing BMDC (Figure 2A). The transduction efficiency increased as multiplicity of infection (MOI) range was increased from 1 to 50 (Supplementary Figure 4A). Construct 1827 was able to reduce the expression of CD31 most effectively and was used in all subsequent experiments (Figure 2B). There was no increase in the extent of CD31 downregulation with increasing MOI (Supplementary Figure 4B).

Following transduction with lentiviral constructs there was little change in the numbers of viable cells in the cultures. There was no difference in the number of viable cells in VitD-CD11c^+^BMDC cultures transduced with negative control vector containing a scrambled siRNA compared to vectors encoding CD31 siRNA (Supplementary Figure 4C). Following the transduction with CD31 siRNA lentivirus, CD31 expression was successfully decreased with no significant changes to MHC class II, CD40, CD80, and CD86 expression compared to control vector treated CD11c^+^BMDC (Supplementary Figure 5A).

To probe the functional effects of CD31 expression on VitD-CD11c^+^BMDC, activated and peptide-pulsed VitD-CD11c^+^BMDC transfected with negative control vector containing a scrambled siRNA or CD31 siRNA construct were co-cultured with naïve cognate CD4^+^ T cells. IL-2 production was significantly increased in co-cultures of CD4^+^ T cells and VitD-CD11c^+^BMDC which had CD31 expression reduced by the siRNA encoding vector (Figure 2C). In addition, production of IFN-γ, GM-CSF, and TNF-α was also increased following priming by VitD-CD11c^+^BMDC with reduced CD31 expression (Figure 2C).

### Overexpression of CD31 Expression on Veh-CD11c^+^BMDC Reduces Their Ability to Prime CD4^+^ T Cells

**Figure 3:**
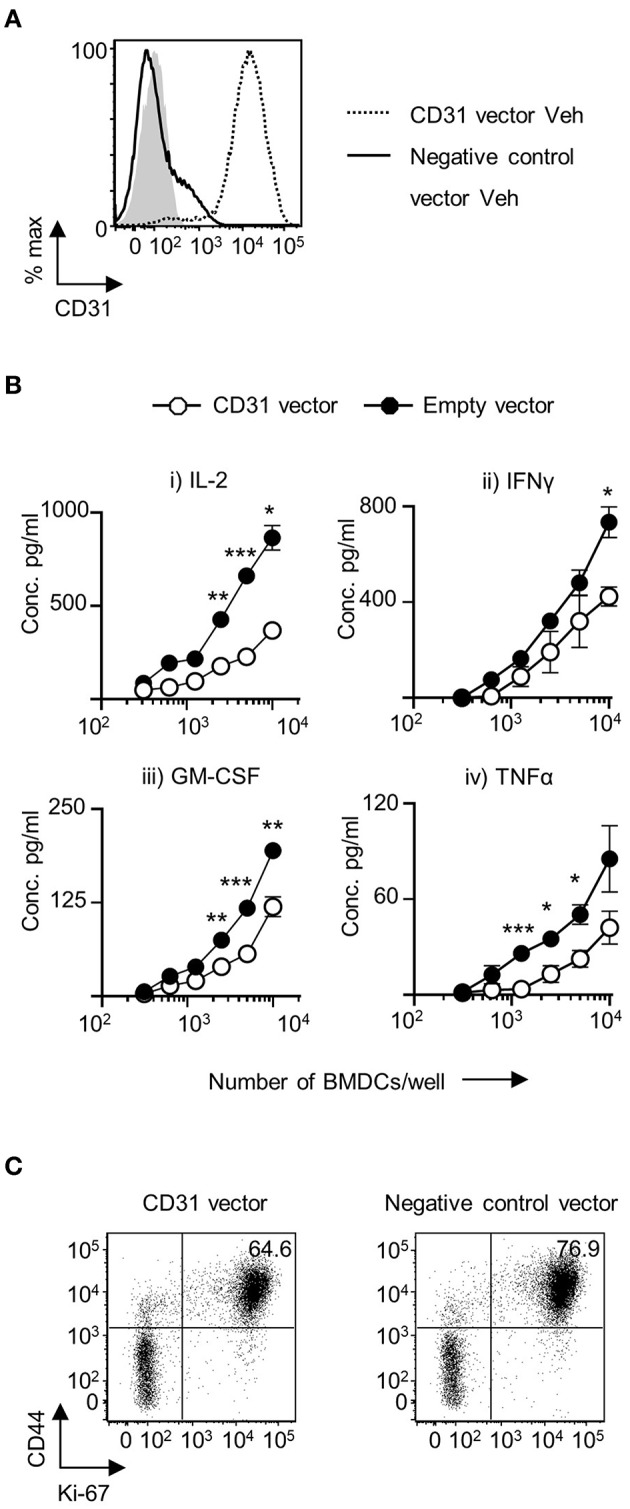
Upregulation of CD31 in VitD BMDC leads to dampened *in vitro* priming of CD4^+^ T cells. Bone marrow derived dendritic cells (BMDC) were generated over 9 days with 20 ng/ml GM-CSF in the absence (Veh) of 1,25(OH)_2_D_3_. On day 6, BMDC were transduced with indicated lentiviral constructs, all expressing GFP. **(A)** CD31 protein expression in GFP^+^ Veh CD11c^+^BMDC transduced with a lentiviral vector expressing CD31 (dotted line) compared to Veh CD11c^+^BMDC transduced with a negative control vector containing a scrambled siRNA (continuous line). Isotype control is indicated by gray shaded area. **(B)** Veh CD11c^+^BMDC transduced with negative control vector containing a scrambled siRNA (black) or CD31 expressing (white) lentiviral vector were matured in the presence of 0.1 μg/ml LPS for 4 h and pulsed with 0.1 μg/ml 4Y peptide, and were subsequently co-cultured at indicated cell numbers with 2 × 10^4^ Tg4 CD4^+^T cells for 48 h (IL-2) or 72 h (all other cytokines). Cytokine release was assessed by ELISA. **(C)** CD4^+^ T cells were analyzed by flow cytometry for CD44 and Ki-67 expression following 72 h of co-culture with indicated Veh CD11c^+^BMDC. *n* = 3, representative of 2 experiments (^*^p < 0.05, ^**^p < 0.01, and ^***^p < 0.001).

To examine the effects of increased CD31 expression on CD11c^+^BMDC, a GFP-CD31 lentiviral construct was developed. The construct was readily incorporated into a proportion of host cells and GFP^+^ cells had a marked increase in CD31 expression (Figure 3A). There was a reduction in IL-2 production by CD4^+^ T cells that were co-cultured with Veh-CD11c^+^BMDC over expressing CD31 (Figure 3B). In addition, pro-inflammatory cytokine production was also reduced from CD4^+^ T cells co-cultured with Veh-CD11c^+^BMDC over expressing CD31 (Figure 3B). The proportion of cells which were dividing and activated was lower in T cells stimulated with BMDC over expressing CD31 (Figure 3C). Lentiviral induction of CD31 overexpression had no influence on the levels of MHC class II, CD40, CD80 and CD86 expression in Veh-CD11c^+^BMDC (Supplementary Figure 5B) or increase the proportion of T cells which expressed Foxp3 (Supplementary Figure 5C). Taken together, the genetic manipulation experiments, which allowed us to both overexpress and downregulate CD31, demonstrated that CD31 on BMDC is a potent regulator of CD4^+^ T cell priming.

### Increased Levels of CD31 on BMDC Reduces BMDC—CD4^+^ T Cell Interaction Time During *in vitro* Priming

**Figure 4:**
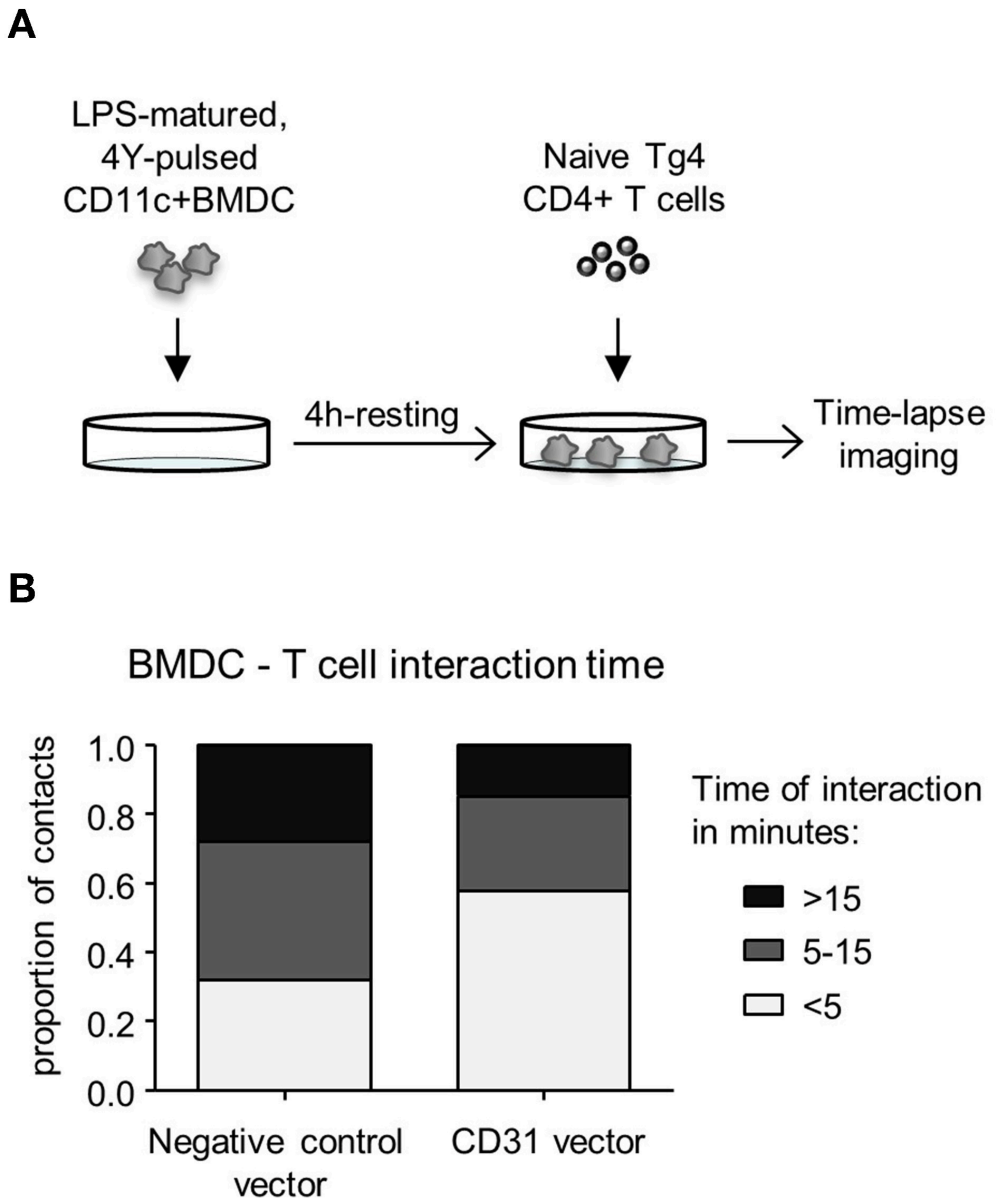
High levels of CD31 on BMDC result in reduced interaction times with CD4^+^ T cells *in vitro*. Bone marrow derived dendritic cells (BMDC) were generated over 9 days with 20 ng/ml GM-CSF in the absence (Veh) of 1,25(OH)_2_D_3_. On day 6, BMDC were transduced with negative control vector containing a scrambled siRNA or CD31 expressing lentiviral constructs, all expressing GFP. **(A)** Experimental layout; on day 9, transduced BMDC were sorted for CD11c^+^GFP^+^ cells and matured for 4 h in the presence of 0.1 μg/ml LPS and pulsed with 0.1 μg/ml 4Y peptide. Matured BMDC were rested for 4 h and Tg4 CD4^+^ T cells added at a 1:4 ratio prior to time-lapse imaging. **(B)** The length of BMDC—T cell interaction was calculated based on images taken in 30 s intervals for the duration of 3 h and were classified as short (< 5 min), intermediate (5–15 min) and long (>15 min). Data pooled from three mice. Representative of 2 experiments.

CD31 has been implicated in mediating cell detachment between live neutrophils and phagocytes, thereby inhibiting binding and engulfment of non-apoptotic neutrophils (29). To understand whether CD31 on DC could likewise favor cell detachment from T cells, Veh-CD11c^+^BMDC were either transduced with negative control vector containing a scrambled siRNA or CD31 overexpressing vector, stimulated with LPS in the presence of cognate antigen, and subsequently co-cultured with naïve CD4^+^ T cells (Figure 4A). CD11c^+^BMDC interaction with T cells was monitored by time lapse imaging and revealed that high CD31 levels on CD11c^+^BMDC led to shorter interaction times with CD4^+^ T cells (*p* < 0.01) (Figure 4B).

### 1,25(OH)_2_D_3_ Enhances CD31 Levels in Human CD11c^+^ Cells

**Figure 5:**
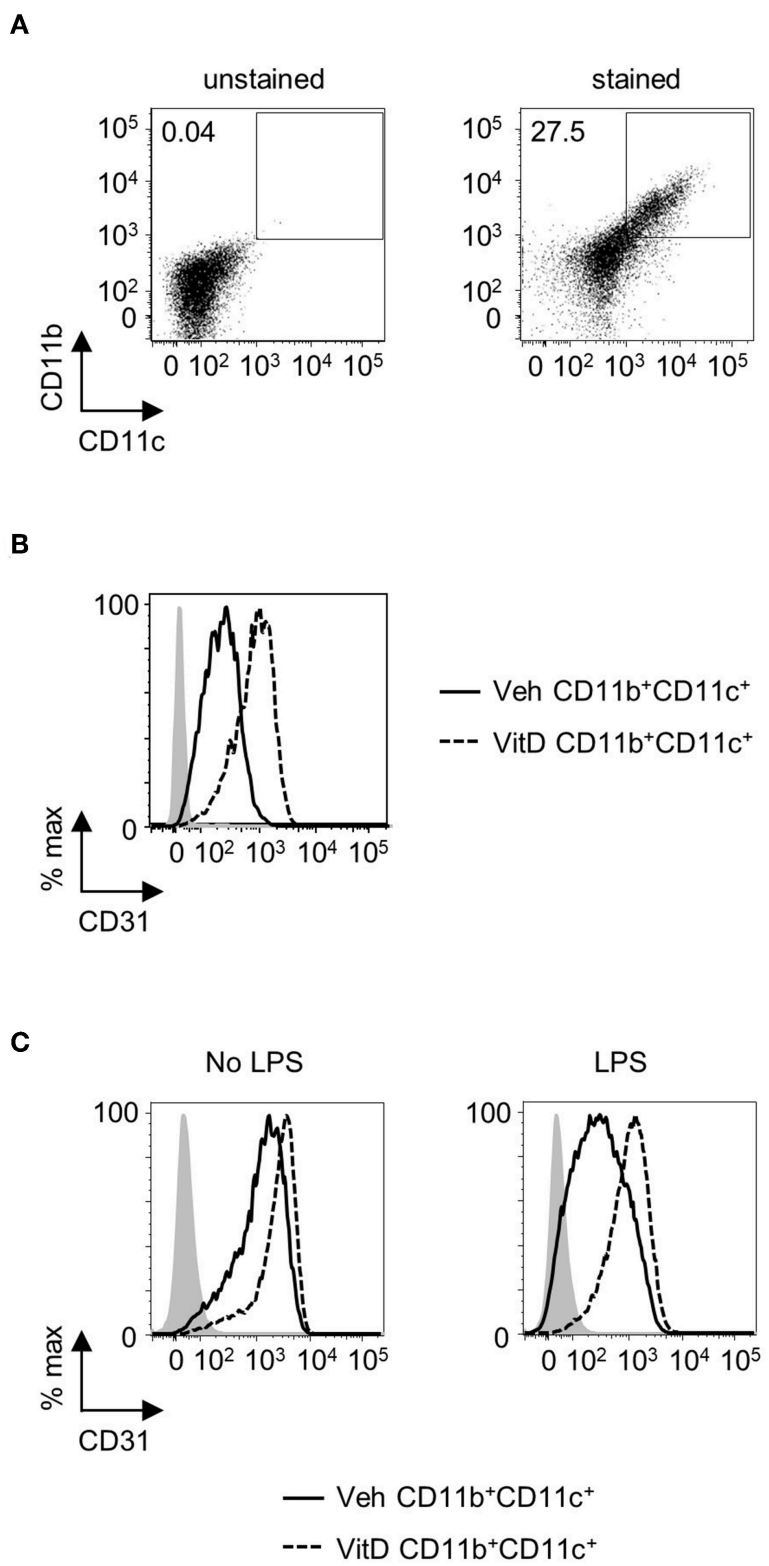
1,25(OH)_2_D_3_ induces elevated levels of CD31 on human CD11b^+^CD11c^+^ cells. Human mobilized blood-derived DC were generated over 7 days in the absence (Veh) or presence (VitD) of 20 nM 1,25(OH)_2_D_3_. **(A)** Representative flow plot of CD11b and CD11c expression on cells after 7 days of culture. **(B)** Representative histogram of CD31 expression in Veh (continuous line) and VitD (dashed line) CD11b^+^CD11c^+^ cells. *n* = 2. **(C)** CD11b^+^CD11c^+^ cells were FACS-sorted and replated with or without 0.1 μg/ml LPS overnight. Flow histograms showing CD31 expression levels in Veh (continuous line) and VitD (dashed line) CD11b^+^CD11c^+^ cells. Isotype control is indicated by gray shaded area.

In order to assess whether 1,25(OH)_2_D_3_ could have a comparable effect on CD31 expression in human DC, CD34^+^ progenitor cells were isolated from mobilized blood and differentiated toward DC in the presence or absence of 1,25(OH)_2_D_3_. CD11b^+^CD11c^+^ cells from 7-day cultures showed a marked increase in CD31 expression when generated in the presence of 1,25(OH)_2_D_3_, suggesting that 1,25(OH)_2_D_3_ could modulate DC function through CD31 not only in mice, but also in humans (Figures 5A,B). When CD11b^+^CD11c^+^ cells were sorted and re-plated overnight with or without LPS, cells generated in the presence of 1,25(OH)_2_D_3_ were able to maintain high CD31 levels whereas control cells showed a considerable reduction in CD31 expression upon stimulation (Figure 5C).

## Discussion

### 1,25(OH)_2_D_3_ Modulates Gene Expression in Immature and Mature BMDC

This study demonstrates that 1,25(OH)_2_D_3_ markedly influences gene expression in CD11c^+^ BMDC, and has unveiled a novel mechanism by which 1,25(OH)_2_D_3_ alters BMDC function—via elevated expression levels of CD31. We focused our approach on studying the effects of 1,25(OH)_2_D_3_ on CD11c^+^BMDC since we have previously established that 1,25(OH)_2_D_3_ leads to the development of a higher proportion of CD11c^−^ cells in GM-CSF-supplemented bone marrow cell cultures (16). Many of the CD11c^−^ cells in 1,25(OH)_2_D_3_ conditioned BMDC do not express cell surface markers such as MHC class II molecules which are considered to be a critical feature of DC (16). Consequently, the gene changes we observed are within the BMDC subset which have the capacity to prime CD4^+^ T cells. Within the hundreds of genes that were differentially expressed in VitD-CD11c^+^BMDC, a range of biochemical pathways were altered when CD11c^+^BMDC were generated in the presence of 1,25(OH)_2_D_3_, as demonstrated in KEGG pathway analysis (data not shown).

An extensive body of work has revealed the dampening effects of 1,25(OH)_2_D_3_ on DC maturation via downregulation of co-stimulatory molecules, MHC-II, and pro-inflammatory cytokines (36). We were therefore particularly interested in genes whose expression was upregulated by 1,25(OH)_2_D_3_, and their functional relevance in DC biology. For further investigation, we focused on genes which were upregulated robustly in both immature and LPS-matured VitD-CD11c^+^BMDC; one of the seven genes fitting this criterion was CD31. CD31 is a member of the Ig superfamily and highly expressed on endothelial cells but also on platelets and the majority of leukocytes (37) and plays an important role in transendothelial migration (38, 39). Beyond its role in cell-cell contact and migration, CD31 has also been found to act as a co-inhibitory molecule on both T cells (21) and DC (30).

### 1,25(OH)_2_D_3_ as a Novel Transcriptional Regulator of CD31 Expression in BMDC

Very little is known about the regulation of CD31 expression in immune cells. In naïve T cells, downregulation of CD31 expression has been observed in association with homeostatic proliferation (40). Human memory T cells, on the other hand, have been reported to acquire CD31 expression following trans-endothelial migration *in vitro* (41). Conversely, in mouse leukocytes, CD31 expression has consistently been found to be reduced upon transmigration through the endothelial cell layer, both *in vitro* (42) and *in vivo* (43, 44), although the details of the mechanisms involved and the functional implications of these expression changes are at present unclear. The data presented here reveal a strong influence of 1,25(OH)_2_D_3_ on the expression levels of CD31 in mouse and human DC and thereby provide a first insight into transcriptional control of this multifunctional glycoprotein. In human DC, CD31 is known to be downregulated upon maturation (45, 46). The reduction in the expression of this co-inhibitory molecule upon DC maturation is thought to allow for an adequate immune response to be mounted upon encounter of infective agents and is likely to play an important role in balancing immune tolerance vs. immunity. In line with this, we observed a downregulation of CD31 expression on human mBDC when matured in the presence of LPS. Strikingly, the presence of 1,25(OH)_2_D_3_ enhanced CD31 levels in immature DC and stabilized CD31 expression during DC maturation with LPS. This suggests that the stably enhanced CD31 expression on human DC through 1,25(OH)_2_D_3_ could contribute to the tolerogenic effects of 1,25(OH)_2_D_3_, as observed in mouse BMDC. Interestingly, we did not observe a reduction in CD31 expression on mouse BMDC matured in the presence of LPS, which does not align with results from Clement et al. (30). In their study, they observe that following LPS stimulation, a subgroup of BMDC that exhibit a more pronounced activation status as defined by higher MHC-II and CD80 expression than the majority of BMDC have slightly lower CD31 expression. However, an overall comparison of CD31 expression between immature and LPS-matured BMDC was not made, and this difference in analysis may account for the distinct observations made. In 1,25(OH)_2_D_3_ conditioned BMDC, however, CD31 expression was greatly enhanced, and high CD31 expression levels were retained upon stimulation with LPS. The details of this 1,25(OH)_2_D_3_-dependent transcriptional control of CD31 expression in mouse and human DC deserve further investigation, and more extensive studies are required to address the transcriptional regulation of CD31 in other cell types which express the Vitamin D receptor and could respond to changes in 1,25(OH)_2_D_3_ concentrations.

### CD31 Expressed by DC Controls DC—T Cell Interaction Time and T Cell Priming

CD31 contains six extracellular Ig C2-type domains. These enable transhomophilic binding of CD31 on adjacent cells (22, 23), but have also been reported to allow heterophilic binding of αVβ3 (25), glycosaminoglycans (26) and CD38 on lymphocytes (27). Mechanistically, we were able to show that high CD31 expression on BMDC led to reduced interaction times with CD4^+^ T cells during *in vitro* priming. This observation is consistent with results published by Brown et al. who elegantly showed that neutrophil CD31 engagement with macrophages induced a detachment signal in neutrophils, thereby avoiding engulfment by the APC (29). Apoptotic neutrophils, however, lacked the intracellular signal and were not able to detach from macrophages, resulting in apoptotic neutrophil engulfment. In the case of BMDC, as presented in the present study, 1,25(OH)_2_D_3_-induced elevated CD31 expression led to reduced interaction time and consequently reduced priming of CD4^+^ T cells. Whether this mechanism involves transhomophilic or heterophilic binding of CD31 has not been established in the present study, but we show that CD31 is expressed on both naïve and activated CD4^+^ T cells. This does not exclude the possibility that CD31 interaction between BMDC and T cells does not also lead to inhibitory downstream signaling in BMDC themselves or T cells in this setting. The cytosolic ITIM domains of CD31, when phosphorylated, recruit Src homology 2 (SH2) domain-containing proteins, e.g., SH2 domain containing protein tyrosine phosphatases SHP-1 and SHP-2 (47). In CD4^+^ T cells, CD31 downstream signaling has been shown by several groups to have inhibitory effects on TCR downstream signaling (35). The reduced activation of T cells during *in vitro* priming with CD31-enriched BMDC may therefore well be the result of a combination of reduced interaction time and CD31 signaling in T cells.

In DC, CD31 has more recently been proposed to act as a co-inhibitory molecule via SHP-1 (30). Clement et al. used a stimulatory CD31 peptide to show that sustained CD31 signaling in DC led to reduced expression of co-stimulatory molecules such as CD40 and CD86, and reduced pro-inflammatory cytokine production (30). In the present study, CD31 expression was modulated, rather than changing its signaling, and altered CD31 expression patterns did not result in changes in surface expression of co-stimulatory molecules. DC tolerized by culture with 1,25(OH)_2_D_3_ have been shown to induce regulatory T cells, and would therefore be a potential mechanism by which CD31 could confer its tolerogenic effect. However, no increase in Treg induction during *in vitro* priming of T cells with CD31-enriched BMDC was observed in this study. In contrast, Clement et al. reported an increase in the Treg pool *in vivo* when mice were injected with DC matured in the presence of stimulating CD31 peptide, attributed to the enhanced secretion of TGF-β and IL-10 (30). It appears that CD31 signaling induced by a soluble CD31 peptide alters the phenotype and cytokine expression pattern of DC toward a more tolerogenic phenotype, which consequently induce regulatory T cells and have an attenuated ability to prime conventional T cells. The present study shows that the overexpression of CD31 in BMDC *per se*, using lentiviral constructs, does not induce a change in BMDC phenotype. The observed reduction in T cell priming by CD31-enriched BMDC is therefore likely a direct result of CD31 binding and not confounded by an altered BMDC phenotype indirectly leading to changes in T cell priming.

### 1,25(OH)_2_D_3_-Induced CD31 Expression on DC: Implications for Treatment of Autoimmune and Chronic Inflammatory Disease?

The use of tolerogenic DC as cell therapy in autoimmune and chronic inflammatory diseases is considered a potential breakthrough in personalized medicine and antigen-specific therapy (5, 15). Although numerous protocols have been described to generate tolerogenic DC, 1,25(OH)_2_D_3_ is one of the most widely used molecules to generate DC with a tolerogenic phenotype (15). A key attribute of tolerogenic DC is to suppress autopathogenic T cells which may be through the induction of anergy or apoptosis or via the induction of regulatory T cells (5). Prevention of further activation of effector T cells is clearly an important attribute of tolerogenic DC; our study indicates that 1,25(OH)_2_D_3_ mediated increase in CD31 expression may be one way in which 1,25(OH)_2_D_3_ could restrain priming ability in tolerogenic DC used in clinical therapies. Our work suggests that evaluation of CD31 expression on tolerogenic DC may be informative on their priming ability prior to administration to patients. In addition, genetic manipulation of CD31 expression may further improve the safety profile of tolerogenic DC by lessening their capacity to activate effector T cells.

## Conclusion

The factors that control CD31 expression on BMDC have been poorly explored to date. This is the first study to demonstrate that the active vitamin D metabolite 1,25(OH)_2_D_3_ can enhance CD31 expression in both mouse and human DC. CD31 acts as a co-inhibitory molecule and prevents effective T cell priming at least in part by reducing the interaction time between BMDC and naïve T cells. This finding is an important demonstration that the failure of 1,25(OH)_2_D_3_ conditioned BMDC to effectively prime T cells is not simply due to the reduced expression of MHC class II and co-stimulatory molecules such as CD40, CD80, and CD86. Exposure of BMDC during development to 1,25(OH)_2_D_3_ is critical in the upregulation of inhibitory pathways which further restrain the ability of BMDC to prime T cells.

## Data Availability

The datasets generated for this study can be found in Gene Expression Omnibus, GSE114768.

## Author Contributions

LS, IM, and RM designed and analyzed experiments. LS and IM performed experiments. AI, DS, and NK contributed to acquisition of experiments. PB designed and optimized lentiviral constructs. KS and JC designed and performed human cell experiments. LS, IM, and RM interpreted the data. IM and RM wrote the paper. All authors contributed to manuscript revision, read and approved the submitted version.

### Conflict of Interest Statement

The authors declare that the research was conducted in the absence of any commercial or financial relationships that could be construed as a potential conflict of interest.

## Abbreviations

4Tyr: myelin basic protein Ac1-9
BMDC: bone marrow-derived dendritic cells
DC: dendritic cells
ITIM: immunoreceptor tyrosine-based inhibitory motif
LPS: lipopolysaccharide
MBP: myelin basic protein
SH2: Src homology 2
TCR: T cell receptor
Veh: vehicle
VitD: vitamin D.
