# Supplementary figures and images for: 1,25-Dihydroxyvitamin D3 Restrains CD4+ T Cell Priming Ability of CD11c+ Dendritic Cells by Upregulating Expression of CD31
Source: Front Immunol. 2019 Mar 28;10:600. doi: 10.3389/fimmu.2019.00600 (PMC6447667; doi:10.3389/fimmu.2019.00600)

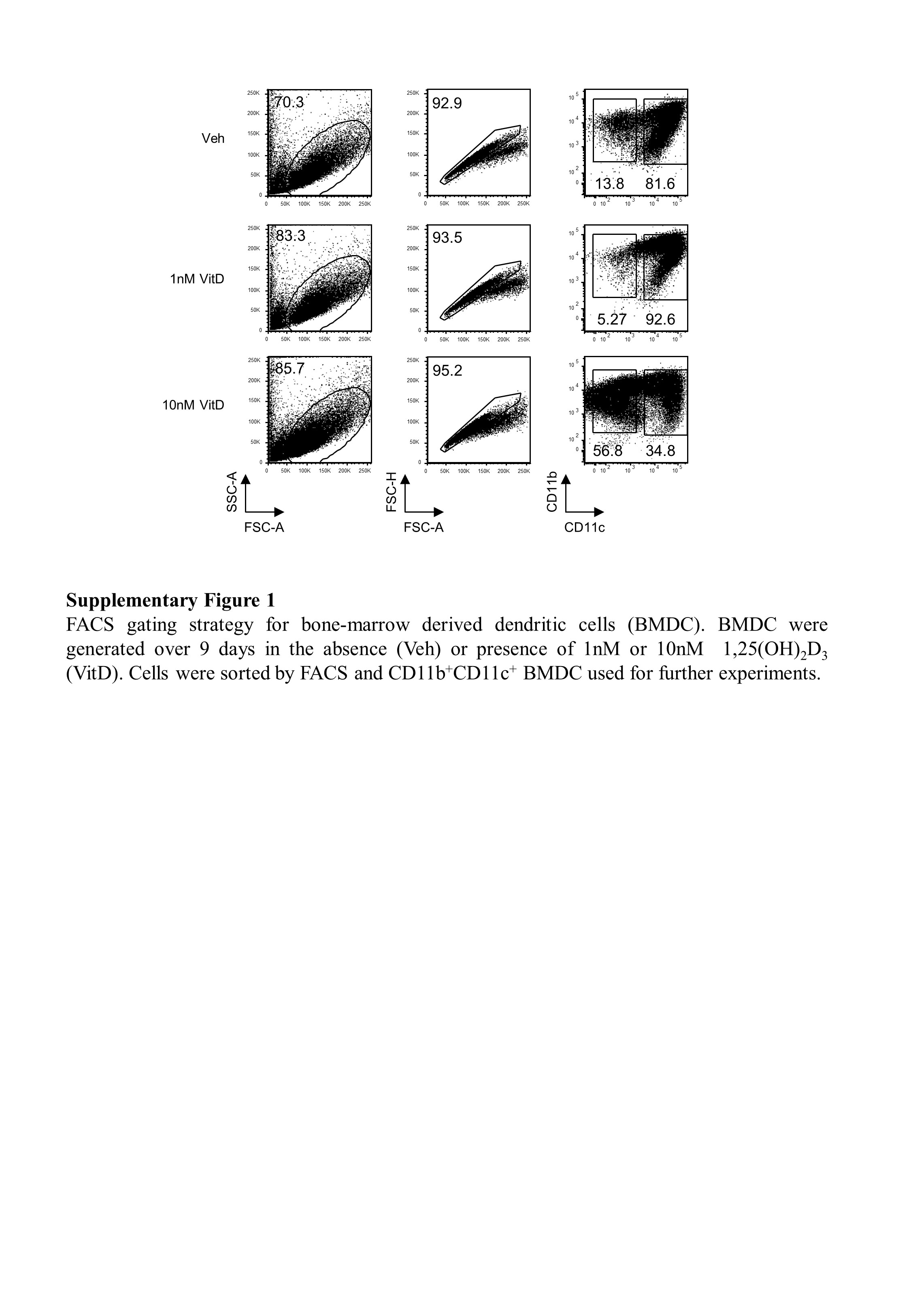

Supplement: Supplementary file 1 [file Image_1.JPEG]

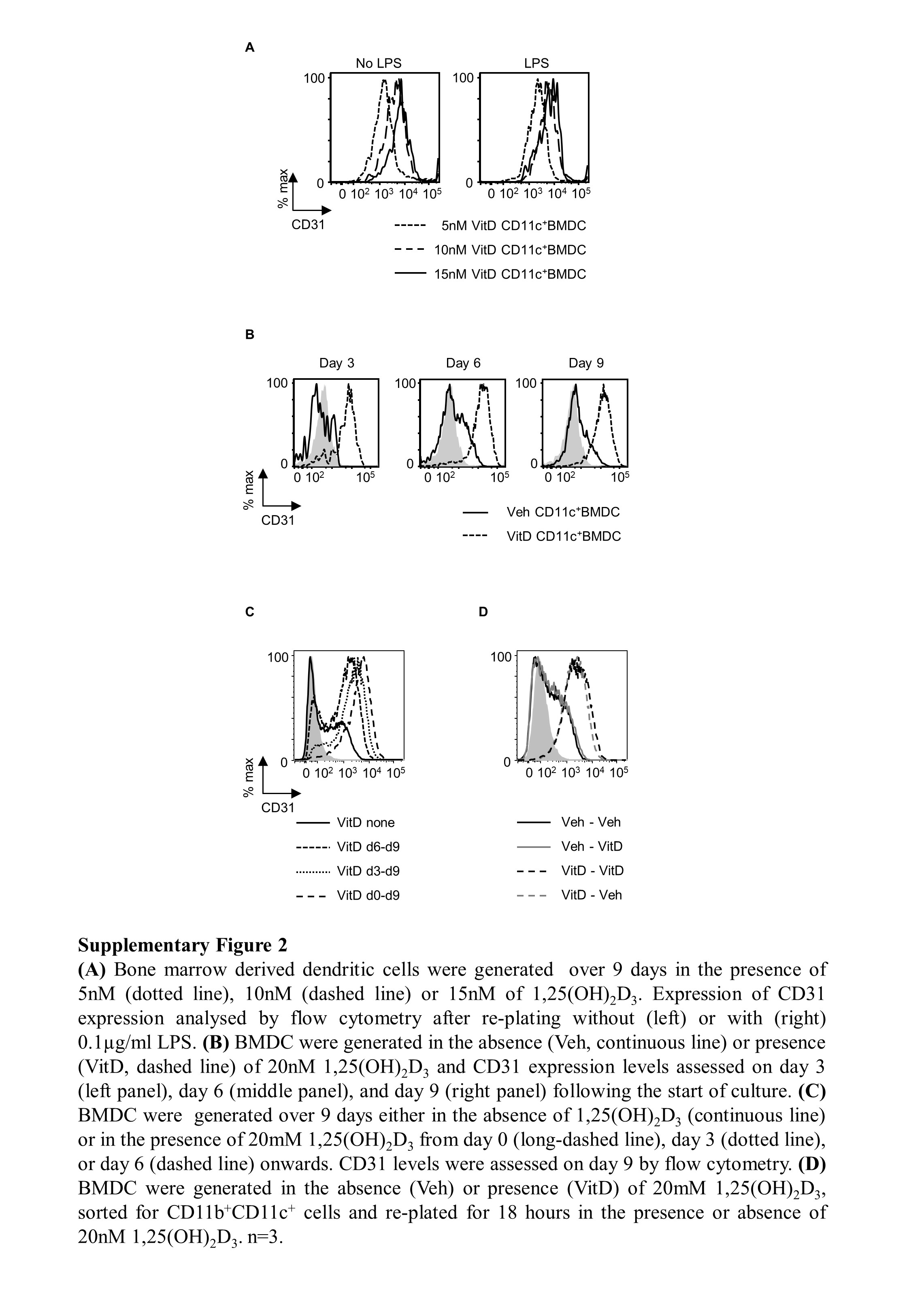

Supplement: Supplementary file 2 [file Image_2.JPEG]

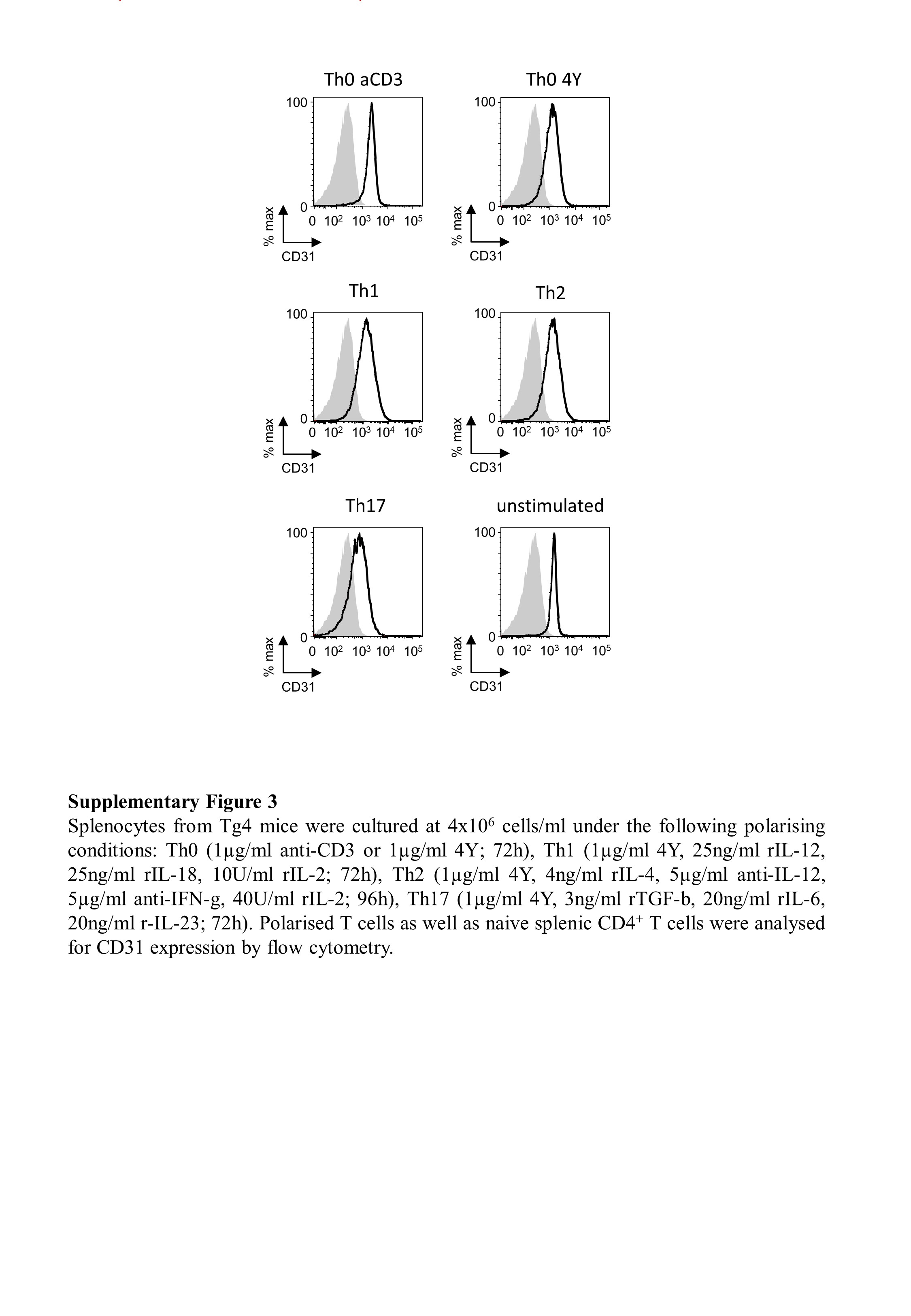

Supplement: Supplementary file 3 [file Image_3.JPEG]

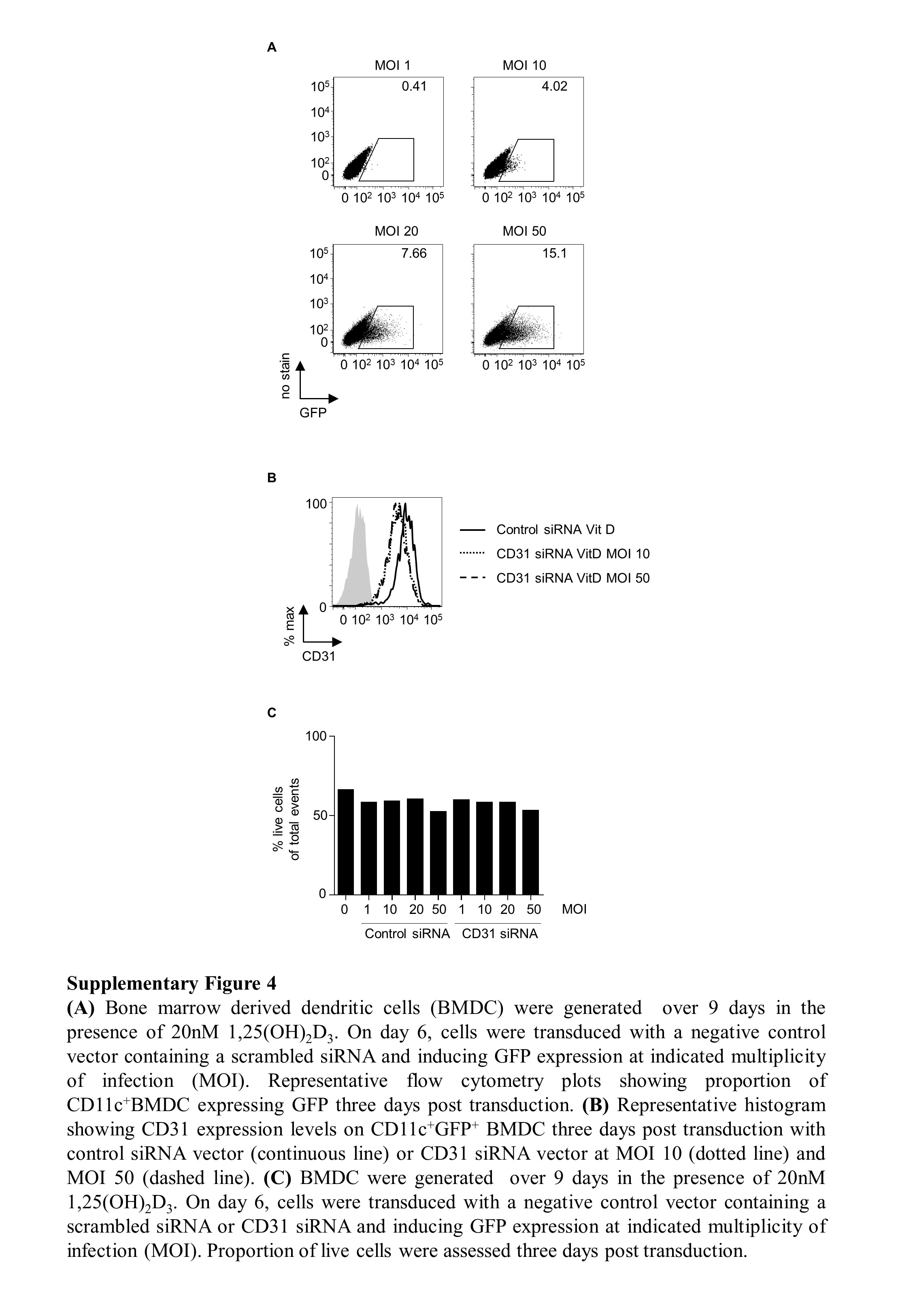

Supplement: Supplementary file 4 [file Image_4.jpg]

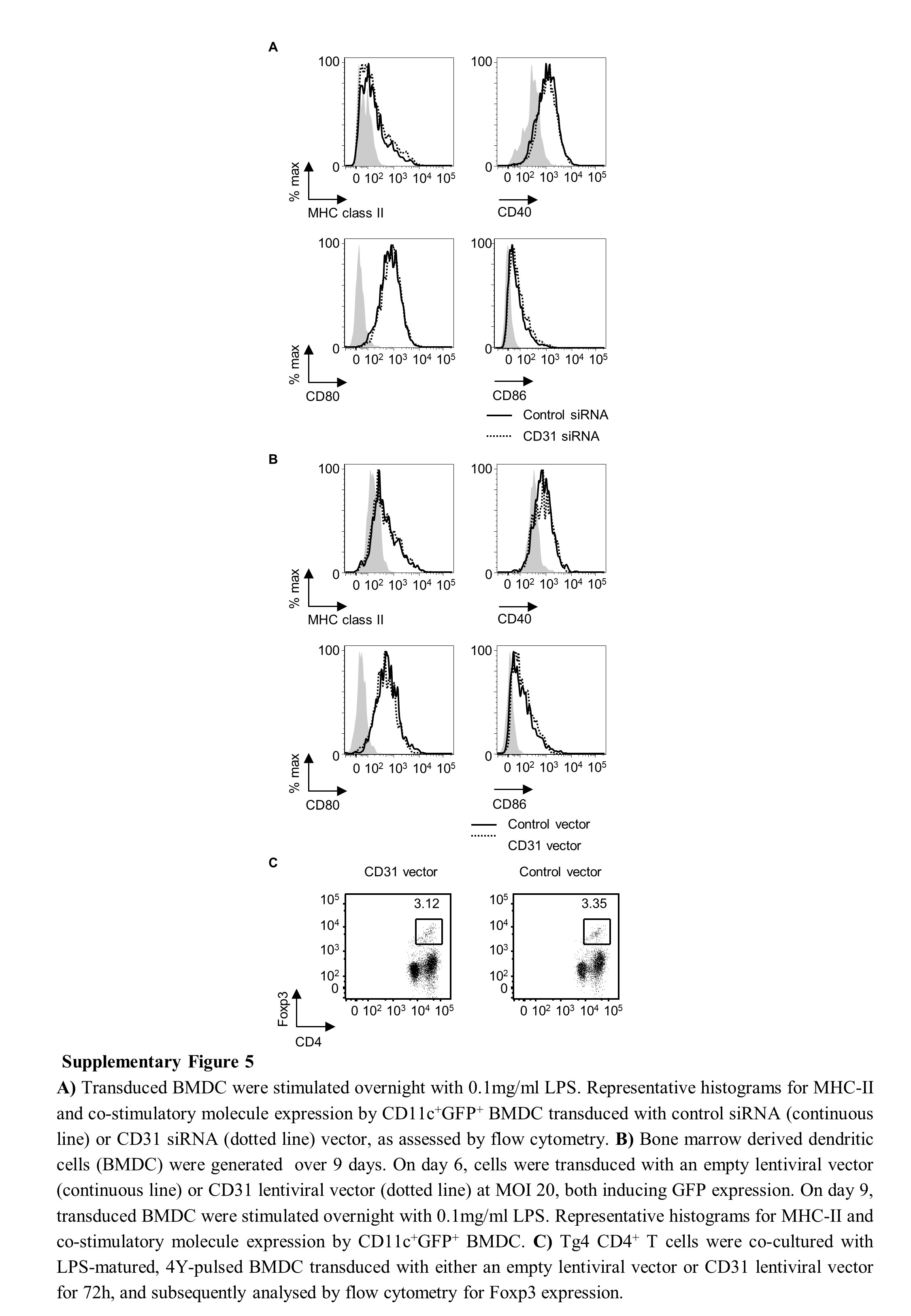

Supplement: Supplementary file 5 [file Image_5.JPEG]
